# Neurogenic Effects of Cell-Free Extracts of Adipose Stem Cells

**DOI:** 10.1371/journal.pone.0148691

**Published:** 2016-02-09

**Authors:** Jae-Jun Ban, Seungwon Yang, Wooseok Im, Manho Kim

**Affiliations:** 1 Department of Neurology, Seoul National University Hospital, Seoul, South Korea; 2 Department of Medicine, University of Chicago, Chicago, IL, United States of America; 3 Protein Metabolism Medical Research Center, College of Medicine, Seoul National University, Seoul, South Korea; Temple University School of Medicine, UNITED STATES

## Abstract

Stem-cell-based therapies are regarded as promising treatments for neurological disorders, and adipose-derived stem cells (ASCs) are a feasible source of clinical application of stem cell. Recent studies have shown that stem cells have a therapeutic potential for use in the treatment of various illnesses through paracrine action. To examine the effects of cell components of ASCs on neural stem cells (NSCs), we treated cell-free extracts of ASCs (CFE-ASCs) containing various components with brain-derived NSCs. To elucidate the effects of CFE-ASCs in NSC proliferation, we treated mouse subventricular zone-derived cultured NSCs with various doses of CFE-ASCs. As a result, CFE-ASCs were found to induce the proliferation of NSCs under conditions of growth factor deprivation in a dose-dependent manner (p<0.01). CFE-ASCs increase the expression of neuron and astrocyte differentiation markers including Tuj-1 (p<0.05) and glial fibrillary acidic protein (p<0.01) without altering the cell’s fate in differentiating NSCs. In addition, treatment with CFE-ASCs induces an increase in neurite numbers (p<0.01) and lengths of NSCs (p<0.05). Furthermore, CFE-ASCs rescue the hydrogen peroxide-induced reduction of NSCs’ viability (p<0.05) and neurite branching (p<0.01). Findings from our study indicate that CFE-ASCs support the survival, proliferation and differentiation of NSCs accompanied with neurite outgrowth, suggesting that CFE-ASCs can modulate neurogenesis in the central nervous system.

## Introduction

Stem cells have been in the spotlight as a novel candidate for use in tissue engineering and regenerative medicine. Among many tissue derived stem cells, adipose stem cells (ASCs) isolated from adipose tissues represent an accessible and abundant source of stem cells with pluripotency [1–3]. Previous studies have demonstrated that ASCs can differentiate into adipogenic [4], myogenic [5–7], angiogenic [8, 9] and neurogenic [10] lineages under appropriate culture conditions. Based on these reasons, ASCs are regarded as potential sources of stem cell transplantation therapy. In addition to direct cell replacement by ASCs, recent studies have reported that stem cells secrete various beneficial factors, which can ameliorate pathological changes. In many reports, this is called a paracrine effect, and secretory factors seem to have anti-apoptotic, angiogenic, wound healing, anti-wrinkle and anti-inflammatory effects [11–15].

In the adult brain, neural stem cells (NSCs) are located in the subventricular zone (SVZ) and dentate gyrus (DG) of the hippocampus and can differentiate into neural cells during adulthood [16–19]. Neurite growth and synaptogenesis are essential and tightly controlled in neuronal development [20, 21], regeneration [22] and plasticity [23]. The functions of NSCs seem to be abnormal in hostile environments in many neurodegenerative diseases [24, 25], and aging seems to reduce the neurogenic potential of NSCs in the brain [26, 27]. Therefore, improvement and/or modulation of NSC functioning are major therapeutic concerns for neuroscientists. However, no research studies have been reported with regard to the therapeutic potentials of cell-free extracts of ASC (CFE-ASCs) containing secretome and their applications to NSCs. Considering these clinical applications, CFE-ASCs could be the most suitable source because it would be possible to show similar effects of stem cell transplantation with no invasive methods and no side effects of cell administration [28, 29].

In this study, we investigated the effects of CFE-ASCs on the physiology of NSCs, including proliferation, differentiation and neuritogenesis. We prepared CFE-ASCs by repeated freeze-thawing and performed in vitro testing including proliferation, differentiation and neurite outgrowth assays using neurosphere cultures. To examine the protective role of CFE-ASCs on oxidative stress, hydrogen peroxide-induced changes in cell viability and neurite growth were examined with or without CFE-ASCs.

## Materials and Methods

### Ethics Statement

This study using human samples was performed with approval from the Institutional Review Board (IRB) of the Seoul National University Hospital. All animal experiments were studied with the approval of the Institutional Animal Care and Use Committee (IACUC, Approval number: 13-0058-C2A1) of Seoul National University Hospital.

### Isolation and culture of human ASCs

Subcutaneous adipose samples were obtained from normal humans who provided written informed consent to participate in the experiment. Adipose tissues obtained from the patients were kept in phosphate buffered saline (PBS) containing antibiotics (Invitrogen, CA, USA) and transported to our laboratory within a day. The adipose samples were digested in 0.075% collagenase type I solution (Invitrogen, CA, USA) with gentle shaking for 1 h at 37°C. Mature adipocyte fractions were removed from stromal fractions by centrifugation at 1,200 × g for 10 min. The remaining stromal fractions were treated with red blood cell lysis buffer (Sigma), for 10 min at room temperature, filtered through a 100μm nylon mesh, and centrifuged at 1,200 × g for 10 min. The remaining stromal fractions of the samples were resuspended and cultured in endothelial growth medium–2 MV (EGM–2 MV; Clonetics, MD, USA), which contained vascular endothelial growth factor, basic fibroblast growth factor (bFGF), epidermal growth factor (EGF), insulin-like growth factor–1, hydrocortisone, and ascorbic acid with 5% fetal bovine serum (FBS). The cells were used for the generation of CFE-ASCs after 3 or 4 passages.

### Preparation of CFE-ASCs

For the preparation of human CFE-ASCs, the cultured ASCs were harvested and centrifuged at 2,000 × g for 8 min after washing twice with PBS. The ASCs (approximately 4 × 10^7^ cells in 175 T-flask) were suspended with 1 ml PBS and lysed by three cycles of rapid freeze/thawing. The lysate was centrifuged at 14,000 × g for 15 min, and the supernatant was passed through a syringe filter unit (0.45 μm). CFE-ASCs were freshly prepared just before treatment. All chemicals were purchased from Sigma. The total protein content of each CFE-ASC was quantified using a bicinchoninic acid protein assay kit (Pierce, IL, USA).

### Mouse NSC culture

We modified a previously published method [30, 31]. To culture primary neurosphere cells, C57BL/6 mice pups, postnatal day-3, were sacrificed by decapitation. Dissections were performed in PBS with 0.6% glucose, pH 7.4, at 4°C using sterile scissor and forceps. For the SVZ dissections, each brain was sliced into 2 mm coronal sections. Tissue was washed in PBS/glucose, digested in papain–dispase–DNase solution (Lorne Laboratories, Twyford, UK); 0.1% dispase (Roche Diagnostics, Hertfordshire, UK) for 4 min, filtered through 40 μm nylon mesh cell filters (Falcon, Suffolk, UK), and then washed three times. Live cells were calculated using the trypan blue dye (Sigma) exclusion method and seeded at a density of 1 x 10^5^ cells/ml in 25 cm^2^ flasks in DMEM/F12 with B27 (Invitrogen, Eugene, Oregon, USA), 1% penicillin /streptomycin/fungizone (Invitrogen, Eugene, Oregon, USA), 20 ng/ml bFGF and 20 ng/ml EGF (R&D Systems, MN, USA).

### NSC proliferation and differentiation assays

To investigate the proliferative effects of CFE-ASCs, we applied CFE-ASCs and 10 μM Bromodeoxyuridine (BrdU, Thermo Fisher Scientific, MA, USA) to cultured NSCs with withdrawal of EGF and bFGF. Two days later, attachment of NSCs to the cover glass in a single cell formation was observed, and NSCs were fixed with 4% paraformaldehyde in PBS. A cell proliferation assay was conducted using CCK-8 reagent (Dojindo, Kumamoto, Japan), according to the manufacturer's instructions. After incubation for 1 hour with WST-1 reagent in a 96-well plate, the absorbance of media was measured at 450 nm (reference at 630 nm) using a Multiskan JX microplate reader (Thermo Lab Systems, MA, USA).

To investigate the effects of differentiating NSCs, passaged neurospheres 150 μm in diameter were transferred to sterile tissue culture tubes and spun at 800 rpm for 5 min. Neurosphere pellets were resuspended with DMEM/F12 with B27 and 5% FBS. Approximately ten neurospheres were then transferred into individual 24-well tissue culture plates containing poly-L-lysine-coated cover glass. After 6 days in vitro, CFE-ASCs were treated for 2 days and cells were fixed with 4% paraformaldehyde in PBS. Immunocytochemistry or microscopic observation was performed to investigate neurite outgrowth. Briefly, to assess neurites numbers and lengths, projected neurites on the central cell body were counted. Each neurite length was measured as the distance of single neurite projection using LAS image analysis (Leica Microsystem, Switzerland). All data was obtained from 5–10 cells and represent to bar graph.

### Immunocytochemistry

Cells fixed on coverslips were treated with blocking solution and permeabilized for 60 min in 4% normal goat serum and 0.2% Triton X-100 in PBS. Primary antibodies for glial fibrillary acidic protein (GFAP) (1:500, Sigma, mouse IgG), Tuj-1 (1:1000, Dako, Carpinteria, CA, rabbit IgG), BrdU (1:200, Pharmingen, rat IgG) were added, and cells were incubated overnight at 4°C. The coverslips were washed in PBS and incubated with secondary antibodies (1:500, phycoerythrin (PE)-conjugated anti-mouse IgG, PE-conjugated anti-rat IgG, fluorescein isothiocyanate (FITC)-conjugated anti-mouse rabbit IgG), and DAPI (Invitrogen, Carlsbad, USA) counter staining was performed. Images were collected using the LSM 510 program on a Zeiss confocal microscope (Carl Zeiss MicroImaging, Inc.). Red and green fluorescence intensities were analyzed by selecting a cellular region of 10–20 cells for each sample and quantified using Nikon NIS elements-AR software.

### Statistical analysis

All values shown in the figures are presented as the mean +/- standard error. Results were analyzed by analysis of variance (ANOVA) followed by post-hoc test or student's t-test. A 2-tailed probability value below 0.05 was considered statistically significant. Data were analyzed using SPSS version 17.0 (SPSS Inc., USA)

## Results

### CFE-ASCs multiply NSCs in the absence of growth factors

**Fig 1:**
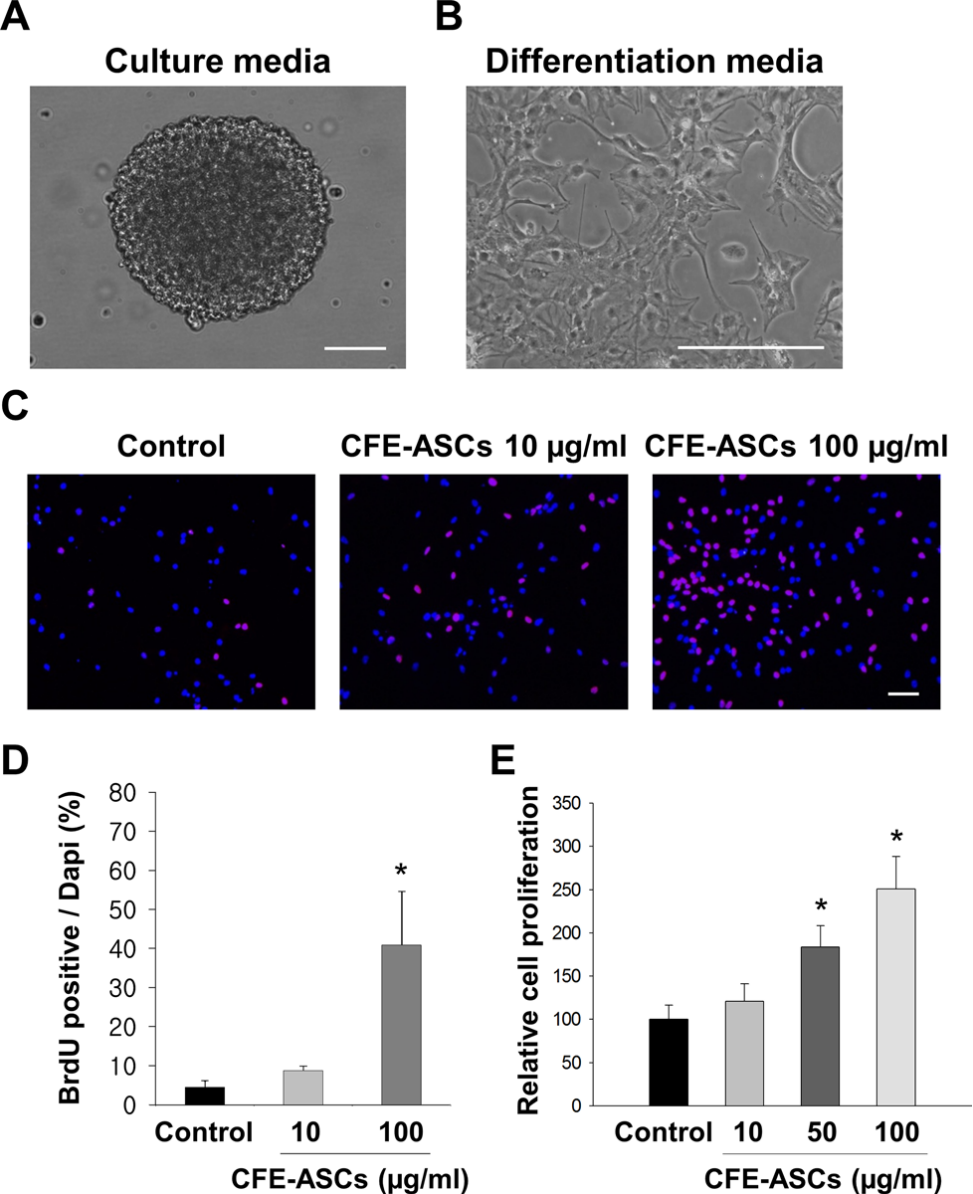
Proliferation of NSC by CFE-ASCs. Primary dissociated SVZ-derived NSC were maintained by the neurosphere method. SVZ tissue was isolated and digested from mice. NSCs were maintained in DMEM/F12/B27 with EGF and bFGF, forming neurospheres (A). After neurosphere cell expansion, these spheres were then transferred into growth-factor-free medium with 5% FBS and kept for 10 days. Without growth factors, spheres were dissociated and attached on coated cover glass (B). SVZ-derived NSCs were treated with CFE-ASCs and BrdU for 2 days in the absence of EGF and bFGF. BrdU (red) and DAPI (blue) staining was performed and observed with fluorescence microscope. Microscopic images showed that CFE-ASCs-treated NSCs have more BudU positive cells than vehicle-treated cells (C). BrdU positive cells were counted and normalized with positive DAPI. Relative cell numbers were represented as bar graphs (n = 5 per group) (D). Cell proliferation assays were performed and relative optical densities were represented as bar graphs (n = 4) (E). *p<0.01 compared with the control group (ANOVA followed by post-hoc test). All data are represented as the mean ± standard deviation (SD). Bar = 50 ìm.

Mouse subventricular-zone-derived neural cells were cultured in DMEM/F12 containing bFGF, EGF, and B27 neuronal medium. Under these conditions, neural stem cells formulated a neurosphere (Fig 1A). After expansion in media containing bFGF and EGF, neuronal stem cells became attached to poly-L-lysine-coated cover glass and expressed differentiation markers within 10 days in the absence of bFGF and EGF (Fig 1B).

We then applied CFE-ASCs and BrdU to the medium with withdrawal of EGF and bFGF. Two days later, NSCs were stained with BrdU and DAPI. As shown in Fig 1C, NSCs maintained with CFE-ASCs had higher BrdU-positive proportions, compared with untreated NSCs. Cell count result of BrdU-positive cells normalized with DAPI positive cells showed that the CFE-ASC-treated group had a higher BrdU positive percentage compared with the untreated group (Fig 1D; p<0.01; n = 5 for each group, ANOVA followed by post-hoc test; control: 4.6 ± 1.5%, CFE-ASC 100 μg/ml: 41 ± 13.8%). The cell proliferation assay using CCK-8 also showed a higher rate of proliferation in ASC-extract treated NSCs (50 μg/ml: 183.4 ± 24.5%, 100 μg/ml: 250.7 ± 37.4% compared with control) than in vehicle treated NSCs (Fig 1E; p<0.01; n = 4, ANOVA followed by post-hoc test). These data explain how CFE-ASCs could maintain NSCs under conditions of growth factor deprivation and contain proliferation factors for NSCs.

### CFE-ASCs promote differentiation marker expression in NSCs

**Fig 2:**
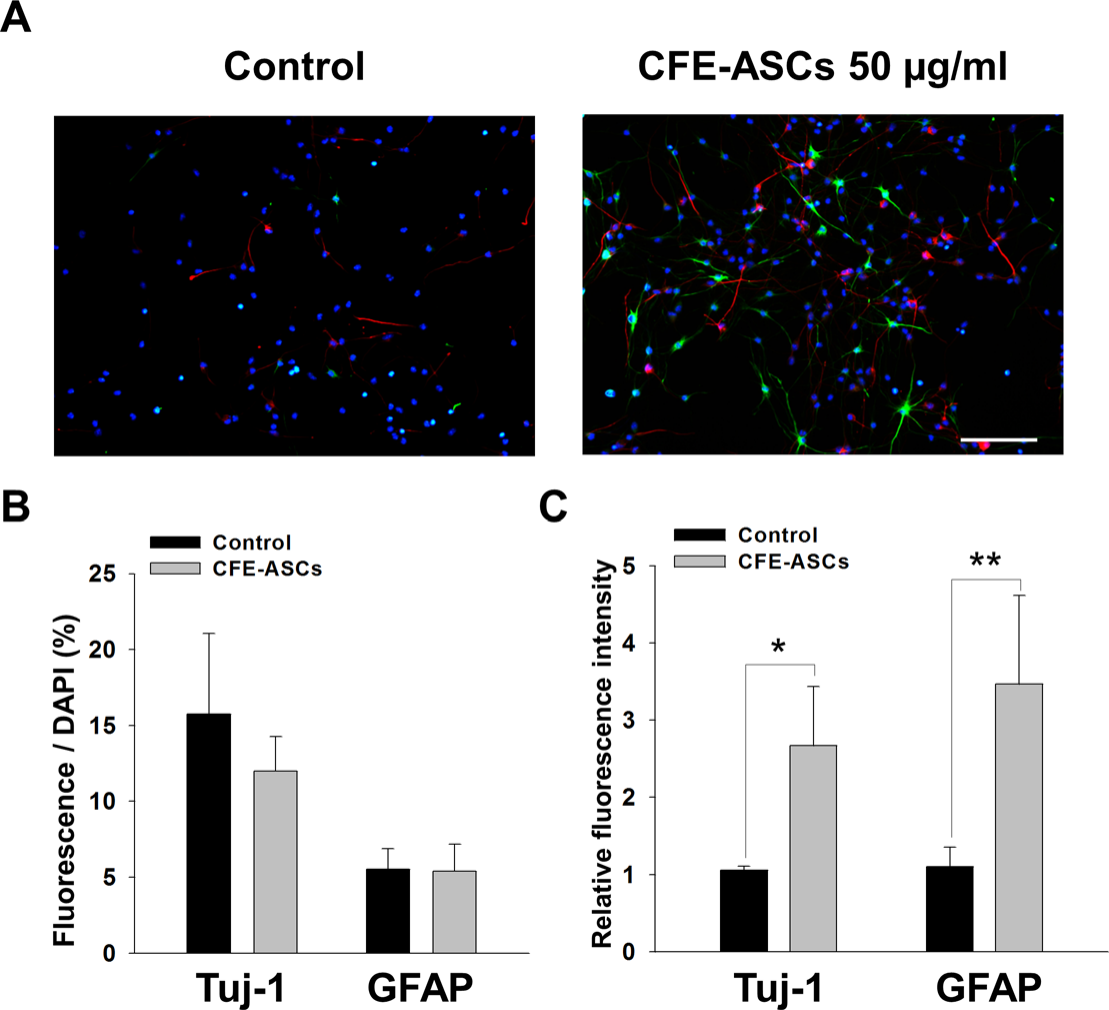
Neural-differentiation promoting effects of CFE-ASCs. NSCs were cultured with DMEM/F12 with B27 and 5% FBS without EGF and bFGF. After 6 days, NSCs were treated with CFE-ASCs or vehicle for 2 days and stained with Tuj-1, GFAP and DAPI. Fluorescence microscopic observation showed expression of Tuj-1 (Red) and GFAP (Green) (A). Positive-stained cells were counted and normalized with DAPI (Blue) count. Vehicle and CFE-ASC-treated NSCs showed no significant differences in positive-cell numbers (n = 5) (B). Fluorescence intensities of Tuj-1 and GFAP were calculated and normalized with DAPI. Relative fluorescence intensity was higher in the CFE-ASC-treated group compared with the vehicle group (n = 5) (C). The data were analyzed using Student’s t-test. All data are represented as the mean ± SD. *p<0.05; **p<0.01 compared with the control group. Bar = 100 ìm.

Without growth factors, NSCs showed spontaneous differentiation and expression of differentiation marker, Tuj-1 or GFAP after 10 days [31]. After culture with NSCs with DMEM/F12 containing B27 and FBS without EGF and bFGF for 6 days, we applied 50 μg/ml CFE-ASCs to attached NSCs for 2 days and stained with Tuj-1, GFAP, and DAPI. Tuj-1, and GFAP, and DAPI positive cells were observed by fluorescence microscope (Fig 2A). We calculated Tuj-1- or GFAP-positive cell numbers normalized by DAPI staining. We observed no significant change in Tuj-1- or GFAP-positive cell numbers after treatment with CFE-ASCs (Fig 2B; n = 5). Relative fluorescence intensities of NSCs with Tuj-1 or GFAP staining were significantly increased by treatment with CFE-ASCs (Fig 2C; Tuj-1: 2.6 ± 0.7-fold, p<0.01; GFAP: 3.4 ± 1.1-fold, p<0.05; n = 5, t-test). These results suggest that CFE-ASCs support the differentiation of NSCs without alteration of cell fates.

### CFE-ASCs promote neurite growth and genesis

**Fig 3:**
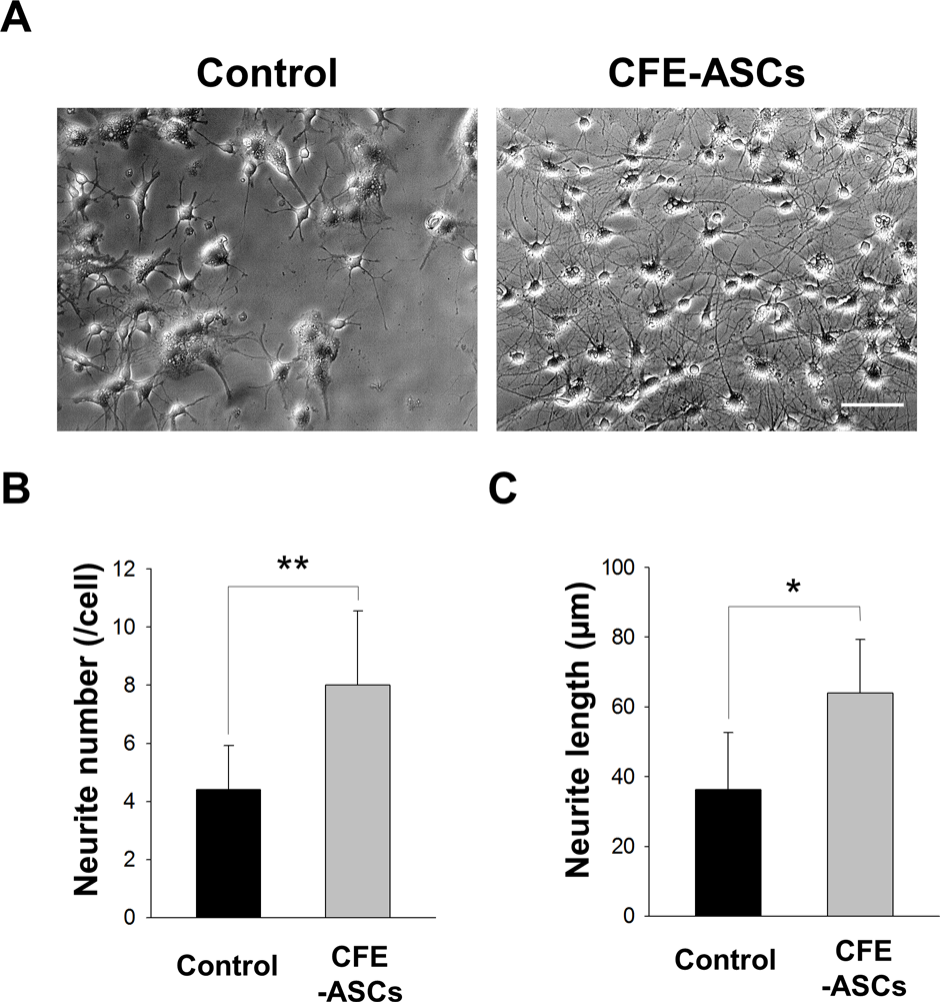
Induction of neurite genesis and growth by CFE-ASCs. Optical microscopic observation was performed 2 days after CFE-ASC or vehicle treatment in differentiating NSCs. CFE-ASC-treated NSCs showed growth and genesis of neurites (A). The numbers and lengths of neurites were analyzed and represented as bar graphs (n = 8 per group) (B). The data were analyzed using Student’s t-test. All data represented as the mean ± SD. *p<0.05; **p<0.01 compared with the control group. Bar = 50 ìm.

To investigate the effects of CFE-ASCs on neurite outgrowth, NSCs were attached in DMEM/F12 containing B27 and 5% FBS for 6 days. We treated these attached NSCs with 50 μg/ml CFE-ASCs for 2 days. Treatment with CFE-ASC resulted in augmented neurite growth and genesis in NSCs, compared with control (Fig 3A). Numbers and lengths of neurites per cell were calculated using LAS Image Analysis (Leica) and represented as a bar graph (Fig 3B; n = 8, t-test). Numbers and lengths of neurites per cell were significantly higher in CFE-ASC-treated NSCs compared with controls (number: 4.4 ± 1.5 vs 8.0 ± 2.5, p<0.01; length: 36.2 ± 16.4 μm vs 64.0 ± 15.2 μm, p<0.05).

### CFE-ASCs prevent hydrogen peroxide-induced neurotoxicity

**Fig 4:**
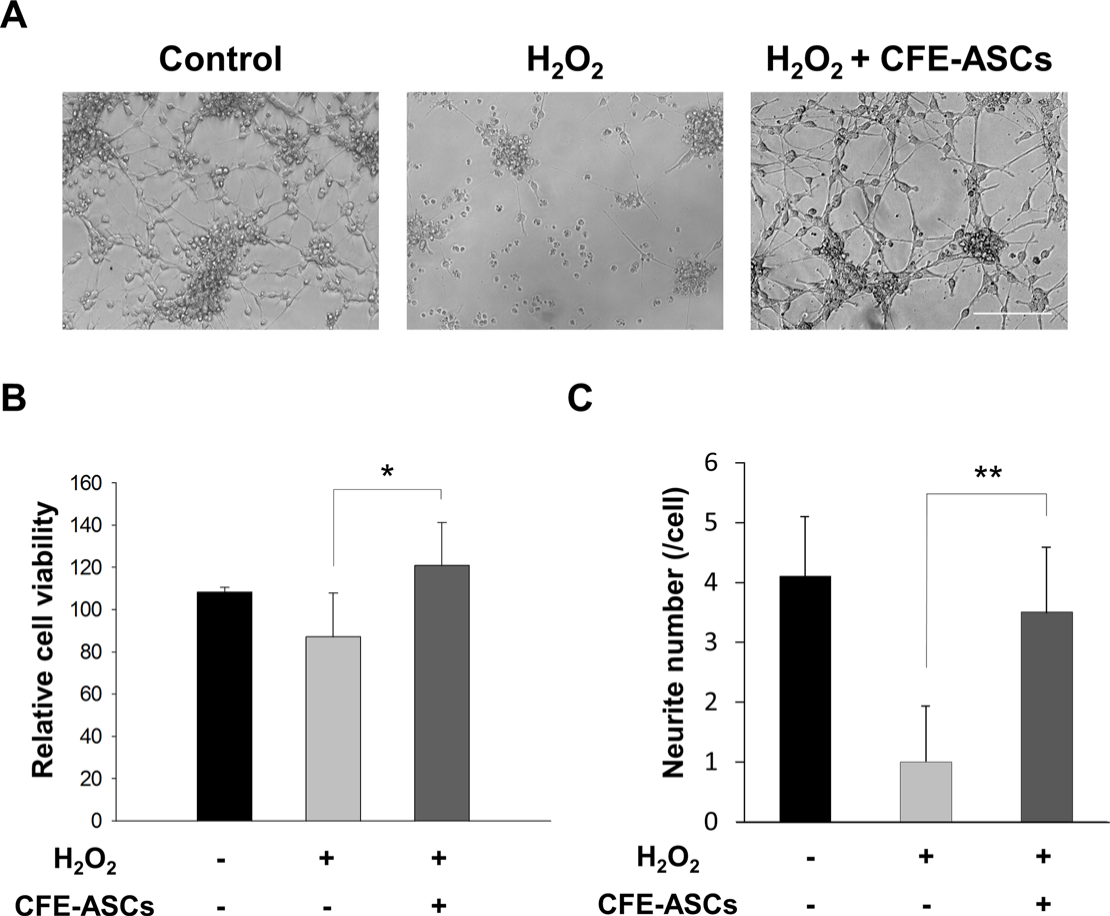
Amelioration of oxidative stress in neural cells. After attachment of NSCs for 7 days in the absence of EGF and bFGF, 10 mM hydrogen peroxide was added to medium with or without CFE-ASC for 2 days, and optical microscopic pictures were obtained (A). The survival rates of the attached neuronal cell population was obtained using WST-1 cell viability assay kits (B). The numbers of neurites were counted and represented as bar graphs. The data were analyzed using Student’s t-test. All data are represented as the mean ± SD. *p<0.05; **p<0.01 compared with the control group. Bar = 50 ìm.

Oxidative stress has a harmful effect in neurological diseases, and protection from oxidative stress is a main concern for disease therapy. Our primary-cultured neural cells were severely damaged in 10 mM hydrogen peroxide for 2 days. We treated neural cells with 10 mM hydrogen peroxide in the presence of 100 μg / ml of CFE-ASCs for 2 days and investigated morphological changes and cell numbers. As shown in Fig 4, the 10 mM hydrogen peroxide group showed severe neuronal death, abnormal morphologies, and a diminishing of the attached cell population. The CFE-ASC-treated group showed an increase in attached neural cells and more intact cell morphology (Fig 4A). The cell viability assay confirmed that CFE-ASC-treated NSCs have higher survival rates than vehicle-treated (Fig 4B, p<0.05), accompanied with a restoration of neurite numbers, which were significantly reduced in hydrogen-peroxide-treated NSCs (Fig 4C, p<0.01). These results imply that ASC-extracts could ameliorate the neuronal damage of oxidative stress in neural cells.

## Discussion

Due to the advantages provided by stem cells, the treatment of disease using stem cells has recently been attracted many attentions. Stem cells are characterized by their capacity for self-renewal and their ability to differentiate along multiple lineage pathways [1]. ASCs are known to have a high proliferation capacity in vitro and the ability to undergo differentiation into multiple lineage cells. As adipose tissue is easily accessible, an autologous application can be performed without immune rejection. Adult neurogenesis is regulated by hormones, growth factors and neurotransmitters, and it is abnormally changed by pathological stimuli such as neurodegenerative diseases, traumatic injury and aging [24, 25, 32, 33]. In addition, aging results in cognitive dysfunction, high susceptibility to neurodegenerative disease and limited recovery of brain injury, and studies have shown that these aging symptoms are closely related to a decline in neurogenic potential in the brain [26, 27, 32]. Thus, NSCs have been regarded as therapeutic targets in many diseases. We used neurosphere assays to investigate the effects of CFE-ASCs on NSCs, and this method provides a useful model of neurogenesis.

We studied the effects of CFE-ASCs in mouse brain-derived NSCs, and we found that cell proliferation was promoted in a dose-dependent manner. Differentiation marker expressions in NSCs were augmented by treatment with extracts. Based on this, we assume that CFE-ASCs could improve the proliferation and differentiation functions of NSCs. CFE-ASCs induce neurite growth and genesis during NSC differentiation. In addition, CFE-ASCs attenuate hydrogen peroxide-induced reductions in viability and neurite numbers in NSCs. These protective roles of CFE-ASCs in NSCs could be beneficial to neuronal injury and neurodegenerative diseases.

Our results suggest that CFE-ASCs could improve NSC proliferation and differentiation in a hostile environment. In many ways, the use of CFE-ASCs is superior to the direct injection of ASCs because it could avoid untargeted differentiation and tumor development after cell administration [28, 29]. CFE-ASCs are easily administered, compared with the injection of ASCs because CFE-ASCs can be easily concentrated and stored. Previous reports have demonstrated the safety and application of extracts of ASCs in a mouse model, suggesting that stem cell extracts can be considered as therapeutic agents [34].

CFE-ASCs also have the limitation of a shorter half-life of their protein components than cell injection. Transplanted stem cells could be maintained for quite a while; on the other hand, protein factors, including neurotrophic factors, have a short half-life, below several hours [35, 36]. Longer half-life proteins and more effective methods could be considered for the efficient clinical application of CFE-ASCs. One possible beneficial factor of CFE-ASCs, extracellular vesicles and/or microRNA, should be further studied [37]. And we can’t observe direct evidence of transport of CFE-ASCs to brain. However, several studies showed that peripheral circulating factors, such as BDNF, IGF or Leptin, can bypass BBB, implying that systemic injection of compounds could affect the CNS [38–41]. Thus, although further examination of in vivo effects and metabolism of CFE-ASCs is essential, our results clearly showed that CFE-ASCs have direct effects on proliferation and differentiation of NSCs.

For further clinical application, in vivo efficacy and safety are required. Previous study has proved safety of systemic injection of cell free extracts of ASCs in rodent model [34], and CFE-ASCs showed no cytotoxic effect on NSCs in vitro (S1 Fig). In addition, the effects of different culture conditions of ASCs should be examined for appropriate culture protocols for better effectiveness.

Findings from our study suggest that, in the treatment of neuronal injury, neurodegenerative diseases and aging symptoms, CFE-ASCs may be a potential resource for non-invasive and cell-free therapeutics. After confirmation of their safe adaptability in humans has been proven, application of autologous human CFE-ASCs may be feasible.

## Supporting Information

S1 Fig Cytotoxicity test of CFE-ASCs. Lactate dehydrogenase (LDH) test was performed 2 days after CFE-ASC or vehicle treatment in differentiating NSCs. Relative percentage of LDH release was calculated using Triton-X100 (TX-100) as 100% (n = 3 per group). CFE-ASCs have no cytotoxicity effect on NSCs. All data represented as the mean ± SD.
(TIF)
