# Supplementary figures and images for: Neurogenic Effects of Cell-Free Extracts of Adipose Stem Cells
Source: PLoS One. 2016 Feb 9;11(2):e0148691. doi: 10.1371/journal.pone.0148691 (PMC4747593; doi:10.1371/journal.pone.0148691)

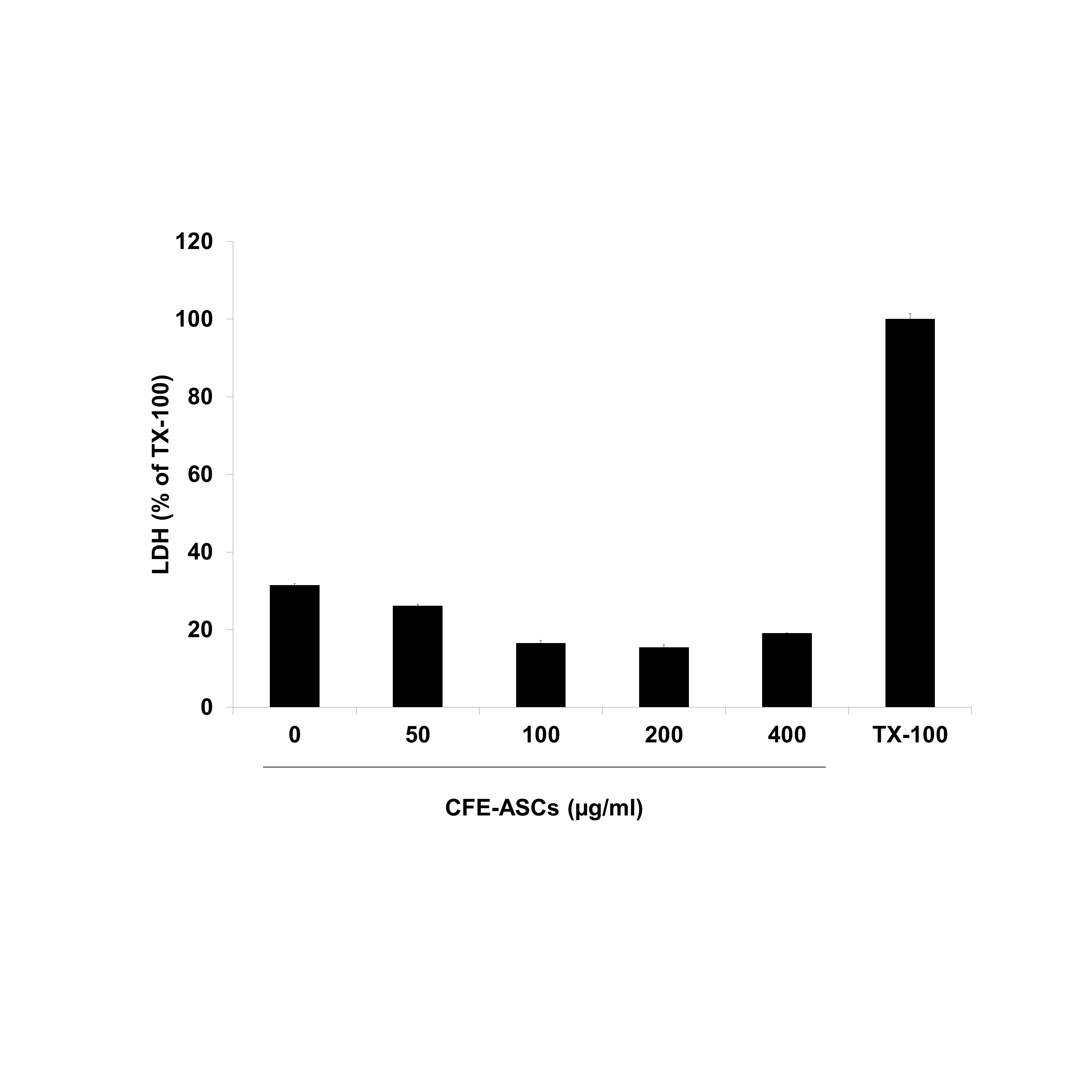

Supplement: S1 Fig — Lactate dehydrogenase (LDH) test was performed 2 days after CFE-ASC or vehicle treatment in differentiating NSCs. Relative percentage of LDH release was calculated using Triton-X100 (TX-100) as 100% (n = 3 per group). CFE-ASCs have no cytotoxicity effect on NSCs. All data represented as the mean ± SD. (TIF) [file pone.0148691.s001.tif]
